# Platelet indices and stroke

**DOI:** 10.1080/0886022X.2018.1456458

**Published:** 2018-04-05

**Authors:** Tarik Yildirim, Fatih Akin, Ibrahim Altun, Seda Elcim Yildirim, Ozcan Basaran, Mustafa Ozcan Soylu

**Affiliations:** Department of Cardiology, Faculty of Medicine, Muğla Sıtkı Koçman University, Mugla, Turkey

We have read with great interest the paper entitled ‘Red blood cell distribution width as a marker of cerebral infarction in hemodialysis patients’ Mo et al. [1] is very important study. We have some suggestions about this trial.

Firstly, platelet functions are affected in chronic renal failure. These patients have abnormal hemostatic status that includes abnormal fibrinogen, circulatuar thromboxane A2 and adenosine diphosphate [2]. Transient ischemic attack and stroke are associated with platelet functions [3]. Thus, platelet functions should be shown in this trial.

Secondly, many investigations recently have shown that mean platelet volume (MPV), plateletcrit (PCT), neutrophyl to lymphocyte ratio are as a predictor of stroke [4]. But there are not any information about this parameters in this trial.

Finally, there are not any data about the patients antiaggregant treatment and patients CHA_2_DS_2_-VASc status. Treatment modality may affect stroke and CHA_2_DS_2_-VASc scoring is an independent predictor of stroke with atrial fibrillation [5].
